# The relationship between external and internal load parameters in 3 × 3 basketball tournaments

**DOI:** 10.1186/s13102-022-00530-1

**Published:** 2022-08-03

**Authors:** C. Willberg, B. Wieland, L. Rettenmaier, M. Behringer, K. Zentgraf

**Affiliations:** 1 Institute of Sport Sciences, Movement Science and Training in Sports, Goethe University Frankfurt, Frankfurt, Germany; 2 Institute of Sport Sciences, Sports Medicine and Exercise Physiology, Goethe University Frankfurt, Frankfurt, Germany

**Keywords:** External load, LPS, Internal load, Team sport, Time-motion analysis, TMG

## Abstract

**Purpose:**

3 × 3 basketball games are characterized by high-intensity accelerations and decelerations, and a high number of changes of direction and jumps. It is played in tournament form with multiple games per day. Therefore, optimal regeneration is crucial for maintaining a high performance level over the course of the tournament. To elucidate how load of a match affects the athletes' bodies (i.e., internal load), muscular responses to the load of 3 × 3 games were analyzed. We aimed to investigate changes in contractility of the m. rectus femoris (RF) and m. gastrocnemius medialis (GC) in response to the load of single 3 × 3 games and a 3 × 3 tournament.

**Methods:**

Inertial movement analysis was conducted to capture game load in 3 × 3. Changes in contractility were measured using tensiomyography (TMG). During a two-day tournament, TMG measurements were conducted in the morning and after each game. Additionally, off-game performance analysis consisting of jump and change-of-direction (COD) tests was conducted the day before the tournament.

**Results:**

Significant changes of the muscle contractility were found for GC with TMG values being higher in the baseline than in the post-game measurements. In contrast to athletes of the GC group, athletes of the RF group responded with either decreased or increased muscle contractility after a single 3 × 3 game. A significant correlation between external and internal load parameters could not be shown. Concerning off-game performance, significant correlations can be reported for COD test duration, CMJ height and ∆Vc as well as COD test duration and ∆Dm. No systematic changes in muscle contractility were found over the course of the tournament in RF and GC.

**Conclusion:**

The athletes' external 3 × 3 game load and their performance level did not seem to affect muscular contractility after a single 3 × 3 game or a complete 3 × 3 tournament within this investigation. This might indicate that elite athletes can resist external load without relevant local muscular fatigue. With respect to the course of the tournament, it can therefore be concluded that the breaks between games seem to be sufficient to return to the initial level of muscle contractility.

**Supplementary Information:**

The online version contains supplementary material available at 10.1186/s13102-022-00530-1.

## Introduction

Three-on-three (3 × 3) basketball is a sport consisting of numerous accelerations, decelerations, jumps, and changes-of-direction, which occur in about 10 min of game duration [1]. Compared to 5-on-5 basketball, games are organized as tournaments, which results in teams playing several matches per day, usually on two or more consecutive days [2]. Knowing the workload of the games is important to understand sport-specific demands, plan training sessions accordingly, and establish optimal regeneration or preparation routines during tournaments [3, 4]. Avoiding a decline in physical performance is, along with the athletes’ tactical behavior, a key parameter for winning competitions [5, 6]. Therefore, it is crucial to gather information about the load the athletes were imposed, their physiological reactions to this load and their recovery. To address this, load monitoring is frequently used in training and competition, where a distinction is usually made between internal and external load parameters.

External load is the physical work performed during training or competition, whereas internal load represents the individual response to those impacts [7, 8]. With the development of microsensor technology in sport, quantifying load has become more applicable, e.g., daily monitoring of training sessions is feasible [9]. However, there is a wide variety of parameters from which to choose.

For monitoring external load, frequently used parameters are the so-called player load (PL, a parameter that summarizes changes of acceleration in x-, y-, and z-direction), acceleration (ACC), deceleration (DEC), distance traveled, and jump count [7–12]. An analysis of external load during international tournaments in 3 × 3 basketball has previously been conducted, e.g., by Montgomery and Maloney [1]. The authors reported that 17% of males’ and 13% of females' total ACC are high-speed movements, defined as ACC with more than 3.5 m*s^−2^. The number of DEC exceeds the number of ACC in moderate (> 2.5 m*s^−2^) and high-speed movements in males and females [1, 13]. Within a single 3 × 3 game, female athletes perform 33.53 (± 13.76) DEC and 32.75 ACC (± 13.85), while male athletes perform 44.16 (± 18) DEC and 33.92 (± 14.59) ACC. On average, the jump count in male 3 × 3 games is 21.8 (± 8.5), with 1.7 jumps being performed per minute. Female jumps count is 16.6 (± 7.5) jumps per game, and 1.4 jumps are performed per minute [13].

As an internal load parameter, most commonly used are peak heart rate (HR), time spent in so-called “work zones” (e.g., defined by the percentage of the individual maximum HR), or HR averages to measure the athletes' internal load [10, 11, 14]. In 3 × 3, an average HR of about 164 beats per minute is reported for males and females [1]. During a tournament, most of the time is spent in the range of 90–100% of the athletes’ peak HR [2]. In addition to the cardiovascular stress, various tissues that comprise the musculoskeletal system are also mechanically stressed [15]. According to Wang et al. [16], mechanical stressors can lead to tissue damage first, a decrease in function second, and tissue repair third. Since the numerous mechanical loads occurring in 3 × 3 basketball are expected to affect the muscular and tendon structures of the athletes, it might also be highly relevant to assess the internal load on this system.

A method to capture internal load caused by biomechanical stress by measuring muscle contractility is tensiomyography (TMG). TMG is a non-invasive method using a single electrical stimulus to assess maximal radial displacement of the muscle belly (Dm) and contraction velocity (Vc). The mechanical reaction of the muscle to an electrical stimulus is a sensitive method for finding changes in muscle contractility before changes in muscle architecture [17]. Increasing Dm and Vc following intense voluntary muscle contraction might be interpreted as post-activation potentiation (PAP; [18, 19]). The underpinning effect is caused by an increase in calcium sensitivity of the actin-myosin complex due to phosphorylation of the myosin regulatory light chain [18]. Decreasing contractility of a muscle, which is accompanied by loss of performance [20–22], can be explained by intracellular accumulation of inorganic phosphate [20]. Accordingly, TMG data can be used as an indicator of the current contractility of the muscle. Increased TMG levels can be caused either by muscle activation or muscular mechanics changes. TMG has previously been used to measure muscular reactions on mechanical load. Both, a decrease in contractility due to high loads [23, 24] and potentiation of contractility have been reported.

However, there are some difficulties when conducting TMG measurements. First, the result of a TMG measurement depends not only on the load but also on athletes’ state [25] since highly trained athletes are supposed to respond differently to external load than untrained subjects. Therefore, explosive-body-performance tests are recommended to get more information on the subjects’ athletic state [26]. Second, especially when there is a limited amount of time for the measurements, there is a need to define the most relevant muscle. Hamner et al. [27] reported the quadriceps muscle group as the largest contributor to braking during the early part of the stance phase and the musculus gastrocnemius medialis (GC) to predominantly contribute during the second phase of braking. Since DEC count is high in 3 × 3, it is assumed that changes in contractility can be analyzed within the musculus rectus femoris (RF) and GC. Also, change-of-direction (COD) movements are assumed to have an impact on muscle contractility (especially of RF) since they result in DEC and consequently eccentric contractions when braking the center of mass to initiate a directional change [28].

Three-on-three basketball is a sport which is characterized by high external loads, where athletes need to resist a high amount of eccentric actions and CODs during a game. To the best of our knowledge, the internal load has yet only been quantified by HR assessments, which reflect the load on the cardiovascular system but not on the musculoskeletal system. Therefore, in this study, we aimed to assess load on the musculoskeletal system by measuring acute muscular response to a single 3 × 3 match and additionally the progress over several games during a tournament, i.e., in a real and applied setting. We hypothesized decreased contractility in GC and RF due to high external load associated with 3 × 3 games.

## Methods

### Participants

Twenty-eight male elite 3 × 3 athletes (age: 27 ± 4.4 years; height: 194.1 ± 5.7 cm; weight: 98.1 ± 9.8 kg), all competing on an international level, and being part of the national 3 × 3 team, participated in the study. Each participant provided written consent for participation in the study, which was approved by the local ethics committee (Grant Number: 2021-30). Due to time constraints based on the tournament mode, TMG measurements were conducted either on GC or on RF. Athletes were randomly assigned to the GC or RF groups. The group allocation of one athlete was changed between the days of the tournament since the TMG data could not be measured reliably on the GC for this athlete. TMG data were visually inspected for each athlete to increase reliability. For this purpose, the individual TMG curves of the athletes were compared for each measurement. In case of atypical curves, e.g., by co-contractions of the deep musculature, the data of the athletes were excluded from analysis. This occurred more often in GC group, which is why the group size of GC group was smaller than the RF group. For this reason, 23 athletes were included in the final analysis. Anthropometric data are shown in Table 1.

**Table 1:** Descriptive statistics of the group

	*N*	Minimum	Maximum	Mean	*SD*
GC					
Age (years)	9	23	34	27.7	4.1
Height (cm)	9	184.5	199.3	191.6	5.1
Weight (kg)	9	83.5	109	95.5	8.2
Arm span (cm)	9	192	213.6	199.5	6.2
RF					
Age (years)	14	22	34	26.6	4.6
Height (cm)	14	187	205	195.8	5.6
Weight (kg)	14	82.1	118.2	99.8	10.7
Arm span (cm)	14	195	213	203.9	6.3

SD, standard deviation; GC, Musculus Gastrocnemius medialis; RF, Musculus Rectus femoris

### Measurement setup

Data were acquired at a two-day international tournament (Prime Heidelberg, Feb. 2021). To measure the subjects' athletic state, a COD test (HAST, [29]), drop jumps (DJ), and countermovement jumps (CMJ) were conducted on the day before the tournament. As a COD test, the so-called Handball Agility-specific test (HAST) [29], consisting of five CODs and including forward–backward running was administered. The HAST is set up as a 5 m square, where three cones (25 cm height) are placed on the top left, top right and bottom right corners and one cone marking the middle of the square. Athletes are asked to perform a start without external start signal. The starting line was set 1 m behind the light barrier (FITLIGHT® Corp., Ontario, Canada), marking the bottom left corner. After the run up, the first COD needs to be performed in the top left corner (touching the cone on the top with the left hand). From there, athletes had to touch the middle cone with the right hand and the top right cone also. Then, athletes run straight back to touch the bottom right cone with their right hand. The middle cone then has to be touched with the left hand and finally shuffle backwards to cross the starting light border. The time for test completion was taken via light gates and then included into analysis.

In the CMJ test, the athletes are asked to jump as high as possible, starting from an upright position and keeping their hands on their lateral iliac crest during the entire movement. Their landing behavior was controlled in terms of resemblance to the concentric action (e.g., no initial landing contact with bend knees). Jumping height was calculated using flight time which was measured via a contact plate (Haynl Elektronik GmbH, Schönbeck).

This system was also used to assess DJ height and contact time. Here, athletes are asked to drop off a box (24 cm height) and jump as high as possible in a reactive manner (i.e., keeping the ground contact time as short as possible).

Standardized instructions is given for all tests. Athletes performed one test trial for familiarization purposes. Afterwards, they performed three trials for each test with a minimum break of 30 s. The value of the best trial (shortest COD test duration/ best reactive strength Index (RSI)/highest jump height) was included in the analysis. The RSI was calculated by dividing jumping height (cm) by ground contact time (ms).External load parameters were measured using a local positioning system (Catapult Clearsky, Catapult Sports, Melbourne, Australia). Twenty anchor nodes were installed at the venues according to the manufacturer’s recommendations. Spatial calibration was conducted using a tachymeter (Leica TS06 Total Station, Leica Geosystems AG, Switzerland). Utilizing a narrow UWB frequency (3.1–10.6 Hz), the system can locate receiver tags (Vector 7, Catapult Sports, Melbourne, Australia) in the surveyed area. Full-court coverage was tested before each measurement. Via Ethernet cabling, the master anchor was connected with the data processing laptop, allowing life tracking and tagging with a 10 Hz frequency. Data were processed using the manufacturer's software (Openfield™ version 3.3.0, Catapult Sports, Melbourne, Australia). Vector 7 receiver tags (81 mm length, 43.5 mm width, 15.9 mm thickness) were attached at the upper back, between the athletes' shoulders using Vector Elite Vest (Catapult Sports, Melbourne, Australia) which allows ECG heart rate analysis due to embedded HR sensors. A 3D-accelerometer (± 16 G, 100 Hz), a magnetometer (− D ± 4900 µT, 100 Hz), and a gyroscope (− 2000 degrees per sec, 100 Hz) are built into the receiver, allowing inertial movement analysis. To gain more information about internal load, athletes were asked to rate their perceived exhaustion per session (session RPE) using a Borg-scale [30] 15–30 min after the end of the game.

For the TMG measurement (TMG; TMG-BMC Ltd., Ljubljana, Slovenia), a high-precision (4 μm) displacement sensor with a spring constant of 0.17 N*mm^−1^ [31] was placed perpendicularly on the muscle belly. The sensor measures the radial displacement over time of a muscle and transfers these data into a digital signal [32]. The locations of the sensor and electrodes were based on the suggestion by Perotto and Delagi [33]. Briefly, for the position of the sensor on the RF, the Spina iliaca anterior superior, as well as the middle of the upper edge of the patella was palpated. A tape measure was then used to determine the midpoint between these two points. The location was marked with a waterproof pen to always measure the same location throughout the tournament [34]. When necessary, the sensor position was slightly adjusted to measure the largest possible point of the muscle belly [35]. An inter-electrode distance of 5 cm was used for the position of the electrodes (self-adhesive; axion, 4 × 4 cm) [36]. Subjects lay in a supine position on an examination couch with the TMG cushion for thigh measurements placed under the right leg to ensure a knee angle of 120° [37]. Similarly, the position of the sensor of the GC measurement was determined according to Perotto and Delagi [33]. The medial femoral condyle was palpated, followed by asking the subject to plantarflex the foot with the knee extended to determine the lower beginning of the muscle belly. The center of these two points defined the position of the sensor. Again, an inter-electrode distance of 5 cm was used. For the measurement of the GC, the subjects lay in a prone position on the examination couch, with the TMG cushion for the measurements of the lower leg placed below the ankle joint in such a way that a pretension of the muscle was given. To trigger the mechanical response of the respective muscle, a single monophasic square wave with a 1 ms pulse was delivered from the TMG stimulator. Since the measurements were taken during basketball competitions, the measurement protocol had to be adjusted due to time constraints. Therefore, only one of the two muscles of the right half of the body was measured from each player. In addition, comparatively large jumps in pulse amplitude were chosen (60 mA, 80 mA, and 100 mA) and measured at 30 s intervals to save time. Randomly, the athletes were allocated to the groups of GC or RF.

**Fig. 1:**
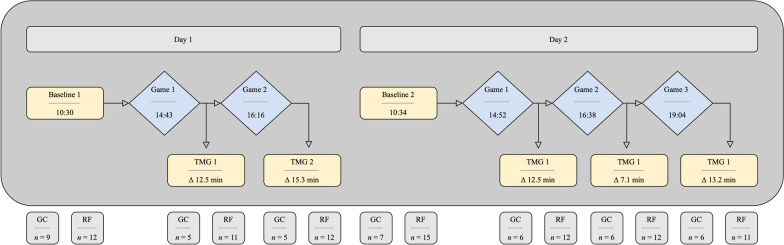
Study design. TMG measurements are marked yellow (square), games of the tournament are marked blue (rough). The time delay of TMG measurements = time of TMG measurement − time of the end of the related game. GC, M. Gastrocnemius medialis; RF, M. Rectus femoris, *n* = count of athletes being taken into analysis

On each day, a baseline measurement was conducted in the morning before any training activity occurred. During the tournament, TMG was set up in a separate space near the court site. After each match, the athletes were asked to get there as quickly as possible, with the athletes of a team always being tested in the same order to maintain approximately the same time between the end of the match and the TMG measurement throughout the tournament. Therefore, each athlete was measured up to seven times within two days (see Fig. 1). As described above, some TMG data had to be excluded after visual inspection which occurred more often in GC than in RF group. For this reason, the count of the athletes being included into analysis in each TMG measurement (*n*) differs between the timepoints of measurements. Since one athlete showed unreliable values of the GC on day one, the group allocation was changed. For this reason, 15 athletes were included in the analysis of the second baseline measurement in RF group. The average time delay between the end of the game and the TMG measurement is 19:18 (± 04:45) min (also displayed in Fig. 1).

### Data processing

Openfield™ was used to process positional and inertial movement data, which were included in the analysis as external load parameters. Player Load © was calculated using the manufacturer’s algorithm (t = time, fwd = forward acceleration, side = sideways acceleration, vert = vertical acceleration).

$$PL= \sqrt{{({fwd}_{t=i+1}-{fwd}_{t=i})}^{2}+ {({side}_{t=i+1}-{side}_{t=i})}^{2}+{({vert}_{t=i+1}-{vert}_{t=i})}^{2}}$$

Inertial movement analysis (IMA) was used to detect PL, jumps and DEC during 3 × 3 games, as those movement reflect eccentric muscular work to a large extent, which is mostly discussed to cause mechanical stress on the musculoskeletal system [38]. Tri-axial accelerometer and gyroscope data (100 Hz) were taken into consideration to evaluate the magnitude of the athlete's movements. To differentiate between athlete and device movement, an advanced gravity filtering model (Kalman filtering technique) was used. IMA ACC contain, based on the direction of sensor movement, positive accelerations, while IMA DEC summarize negative acceleration values.

TMG raw data were saved in Microsoft Excel (Version 16.5, Microsoft Corporation, Redmont, USA) for each athlete. Each measurement was visualized and compared to other trials to exclude possible erroneous measurements. Only the values of the curve with the highest maximum Dm was used for analysis. In addition to Dm, Vc was calculated using MATLAB® R2019b (MathWorks, USA) and was defined as the calculated slope of the displacement curve over time. Vc was calculated as: (90%Dm–10%Dm) / (contraction time from 10 to 90%Dm). To analyze changes of TMG parameters, ∆Dm and ∆Vc were calculated for each game by subtracting post-game values from pre-game values for each athlete in each measurement. Therefore, when ∆Dm and ∆Vc had positive values, there was an increase in contractility which was interpreted as PAP [18, 19], when values were negative, contractility decreased which indicates a loss of performance [20–22].

For analysis of the muscular reactions on the external load of a single 3 × 3 game, athletes were grouped calculating the percentage of change in Dm, since we assumed that athletes might respond in both directions. Group allocations are displayed in Table 2.

**Table 2:** TMG analysis of a single game. Group allocation of all athletes who had a baseline measurement and a TMG measurement after game 1

	Day 1				Day 2			
Group	∆Dm > 0		∆Dm < 0		∆Dm > 0		∆Dm < 0	
Muscle	GC	RF	GC	RF	GC	RF	GC	RF
*N*	1	5	4	4	0	4	6	8

GC, M. Gastrocnemius medialis; RF, M. Rectus femoris

### Statistics

Statistical analyses were performed using IBM SPSS (version 26, IBM Corporation, Armonk, New York). Descriptive statistics included means, standard deviations, minimum, and maximum. Graphs were generated using Microsoft Excel (Version 16.5, Microsoft Corporation, Redmont, USA).

All data were analyzed within the groups (GC and RF). When investigating differences of contractility within a single game, the first games of each day were taken into account since TMG values can be directly compared to the baseline measurement of each day. To analyze, if there are differences in TMG parameters regarding the baseline and the post-game measurements, a 2 × 2 repeated measures ANOVA was calculated including TMG values (Dm, Vc) and timepoint of measurement (pre-, post-game). Focusing on the relation of internal and external load, bivariate correlations (Pearson’s r) were calculated with PL, DEC, jump count, ∆Dm, and ∆Vc. The level of significance was set to α < 0.05. Additionally, a bivariate correlation (Pearson’s r) was calculated including ∆Dm, ∆Vc and the time delay between the end of the game and the TMG measurement.

For further analysis, athletes were grouped according to their muscular load response. This allowed group comparisons, which were conducted using the athletes' performance in jumping and COD tasks. Correlations were calculated including ∆Dm and performance parameters. The results were grouped concerning the muscle analyzed and the muscular response (∆ > 0: positive response, ∆ < 0: negative response). Also, we aimed to describe group differences which muscular responses might be based on.

## Results

The average PL of a single 3 × 3 game is 126.0 (± 35.9) arbitrary units (a.u.). 23.6 (± 7.8) DEC and 21.7 (± 8.4) jumps are performed per game. The average time between the end of the game and the TMG measurement was 19:18 (± 04:45) min. Athletes rated the games as being more and more exhausting as the tournament progressed (5.8 ± 1.6 to 7.3 ± 1.8).

The 2 × 2 ANOVA, showed a significant interaction effect (time*TMG value: F(1,10) = 19.39, *p* = 0.01). Main effects of TMG variables are significant in both, GC (F(1, 10) = 45.63, *p* =  < 0.01; diff_mean_ = 2.1, CI [1.4–1.8]) and RF group (F(1,20) = 212.46, *p* =  < 0.01; diff_mean_ = 7.9, CI [6.7–9]). In GC group, there is also a significant main effect of time (F(1,10) = 20.73, *p* = 0.01). TMG values were higher in the baseline measurement than in the post-game measurement (diff_mean_ = 0.22, CI [0.11–0.33]).

There is no significant correlation between external and internal load parameters (Table 3). Regarding internal load variables ∆Dm and ∆Vc correlate significantly in the RF group (*r* = 0.87, *p* < 0.01). External load parameters also show significant correlations in RF and GC for DEC and PL (GC: *r* = 0.72, *p* = 0.01; RF: *r* = 0.64, *p* < 0.01). Jump count correlates with DEC in GC group (*r* = 0.62, *p* = 0.04). No significant correlations can be reported concerning the time delay and ∆Dm as well as ∆Vc in GC and RF group.

**Table 3:** Correlation matrix of external game load and TMG response following a single 3 × 3 game

	M. Gastrocnemius				M. Rectus femoris			
	DEC	Jumps	∆ Dm	∆ Vc	DEC	Jumps	∆ Dm	∆ Vc
PL								
Pearson	0.72*	0.39	0.07	0.58	0.64*	0.73	− 0.13	− 0.17
*p*	**0.01**	0.23	0.85	0.06	**< 0.01**	0.76	0.57	0.46
*N*	**11**	11	11	11	**20**	20	21	21
DEC								
Pearson		0.62*	0.26	0.30		0.17	− 0.29	− 0.41
*p*		**0.04**	0.45	0.37		0.47	0.21	0.07
*N*		**11**	11	11		20	20	20
Jumps								
Pearson			0.40	0.31			− 0.22	− 0.18
*p*			0.23	0.36			0.35	0.45
*N*			11	11			20	20
∆ Dm								
Pearson				0.51				0.87*
*p*				0.11				**< 0.01**
*N*				11				**21**

Significant correlations (p < 0.05) are marked bold

PL, player load; DEC, deceleration; Jumps, total jump count; GC, M. Gastrocnemius medialis; RF, M. Rectus femoris

Since the athletes' TMG response is independent of game load and is either positive (increasing) or negative (decreasing), performance parameters were analyzed to test if differences in the athletic performance can explain different TMG responses. Significant correlations between TMG response (Dm) and performance test (COD) was only found in RF positive response group, on day two (*r* = − 0.98, *p* = 0.02).

There is only one athlete in the GC group who showed a positive response after playing one 3 × 3 match. Slight differences in the performance parameters of this athlete (higher jumping height and RSI, but longer COD duration) can be shown when comparing the values to the other athletes of the GC group. Within the RF group, 12 athletes showed a negative response, nine showed increased TMG values (see Tables 2, 4). There are small differences between the performance parameters in this group. Since *SD* is larger than the group differences, we do not assume that the athletic state influences contractility of the muscle after a 3 × 3 game.

**Table 4:** Means and standard deviation of the athletes' performance parameters grouped by the muscular response in game one of each day

	*N*	CMJ		DJ		COD	
		Mean	*SD*	Mean	*SD*	Mean	*SD*
GC							
Neg. response	10	46.98	4.09	0.19	0.03	6.91	0.20
Pos. response	1	49.84	0	0.22	0	7.20	0
RF							
Neg. response	12	40.85	5.06	0.16	0.04	7.15	0.20
Pos. response	9	39.84	5.58	0.15	0.05	7.45	0.51

SD, standard deviation; Neg, negative; Pos, positive; GC, M. Gastrocnemius medialis; RF, M. Rectus femoris

Because 3 × 3 is played in a tournament mode, we aimed to report changes in the individual TMG response during the tournament. However, although data were captured in all 23 participants, TMG measurements after every single game could only be acquired in three athletes of the GC group and six athletes of the RF group. Therefore, data of those athletes are presented descriptively in a Additional file 1. There is no clear trend to be reported regarding both, load and TMG parameters. Some athletes rather show potentiation of contractility, some show only small changes during the tournament. A high decrease in contractility can only be seen after single games but not during the course of a game day.

## Discussion

External load, which can be quantified by microsensor technology, leads to reactions of both the cardiovascular and musculoskeletal systems [15]. We aimed to investigate the effect of mainly eccentric load parameters on muscular tissue by analyzing its contractility.

Repeated measurements ANOVA revealed significant effects of time only in the GC group, with TMG values being higher in the baseline than in the post-game measurements. This is in line with the further analysis of the TMG response, where only one athlete showed increased TMG values, while in the RF group, increased and decreased values can be reported with equally high incidence. One possible explanation might be the muscle fiber contribution of GC and RF. Dahmane et al. [39], Staron et al. [40] and Pierrynowski and Morrison [41] reported that there are more type II muscle fibers in the superficial area of the RF than the GC. Due to lower basal calcium sensitivity and myosin light chain kinase activity, the effect of PAP is assumed to be greater in type II fibers than in type I fibers [42–44]. Primarily, PAP is driven by an increase in muscle’s calcium sensitivity due to the phosphorylation of the myosin regulatory light chain [18]. In contrast to PAP mechanisms, muscular fatigue is driven by phosphate accumulation which leads to decreased calcium sensitivity [45] and, therefore, a depression of the contractile response. The question arises whether or to what degree the athletes’ external load is critical to the muscular response. We hypothesized that external load parameters correlate with the muscular response.

There was no significant correlation between internal and external load parameters when analyzing a single 3 × 3 basketball game. In GC, out of 11 athletes, two athletes responded with increased Vc values post game. All others showed decreased Vc values—unexpectedly, the decreases were higher when game load was lower. Concerning the Dm, only one athlete responded positively (higher Dm post game). In contrast to Vc, high external load leads to higher decreases in Dm. However, there is no significant correlation of load parameters between Vc and Dm in GC group, which might be caused by small sample size (especially in GC group) and high variance within the data. Also in the RF group, neither Vc nor Dm were correlated with external load parameters occurring during a 3 × 3 game. While most athletes in the GC group responded with decreased Dm and Vc values, athletes in the RF group showed both increased as well decreased TMG values. Since, in some cases, athletes responded with increased values in one game and decreased values in another game, the question is whether there is an individual threshold after which potentiation turns into muscular fatigue. Rassier and MacIntosh [45] stated that the contractile response is dependent on the amount and intensity of muscular work with brief repetitive stimulations leading to a potentiation of the contractile response and continued stimulations resulting in impaired contractile response (fatigue) which can be explained by the common mechanisms of PAP and fatigue, which are caused by a change in calcium sensitivity [45].

When analyzing the athletic level of the subjects in this study, significant correlations between performance test results and TMG values were only found in the RF group where COD test time correlated with ∆Dm. The athletes who were faster in the COD test showed higher (positive) changes concerning the muscle contractibility. However, sample size is very small for this group, so this can only be interpreted as a small indication that off-game performance might be a factor influencing changes in the TMG response. In the other tasks and groups, there are neither weak, middle or strong correlations to be reported. One reason for the result may be the demands of the tasks given. When performing a COD movement or jumps, not only the muscular response but also intra- and intermuscular coordination and neural mechanisms determine the performance output [46]. Also, especially concerning the COD task, technical aspects, as well as the scores in jumping tests, might have influenced the performance. Concerning the DJ task an association between contact time and Dm in rectus femoris of elite soccer players was reported by [46]. Even though TMG results are moderately related to factors linked with a stretch–shortening cycle related task in their study, the authors state that TMG parameters are no predictors for power-dependent motor tasks [46]. Regarding the results of this investigation, again, there is only small variance within the data, especially regarding the COD test duration. One explanation for the lack of variance might be the expertise level of the athletes tested within this study. All athletes competed at national state level and therefore did perform well in the performance tests. Since no major differences were seen, it could be assumed that due to the high training status, even a 3 × 3 game does not result in any significant changes in the contractility of the musculature. In their review, García-García et al. [47] reported changes in TMG parameters in diverse sports (e.g., endurance athletes, sport games, and gymnastics) provoked by diverse stimuli (training periods, competition, rehabilitation). When analyzing professional soccer and rugby players, altered TMG values were reported for both, Dm and Vc in RF. However, these results were reported after an 8- to 10-week training period or during a season. This raises the question of whether the external load of a single match is not high enough to affect muscle contractility in highly trained athletes.

As described above, one may assume that external load can have a two-way influence on TMG parameters – either showing a potentiation or a decrease in contractility. We would assume the relation of load and muscular response to be of an inverted-U type with an individual optimum load, leading to great PAP and even higher load leading to fatigue. The work of Peterson and Quiggle [48] supports this theory. The authors also investigated the internal–external load response in female basketball players during one entire season using TMG and accelerometer-based load analysis. The authors found significant positive correlations between contraction time and the external load (accumulated count of ACC and DEC in all directions). More interestingly, they stated that a decrease of external load (13%) led to substantial decreases in internal load (20%). Also, an increase in external load (40%) led to an increase in internal load (18%) [48]. However, the sample size in their study was quite low, which is why further analysis has to be conducted. Regarding the data of this investigation, we cannot show proof for an inverted-U-theory since the variance within the load data is small. This might also be the reason for missing correlations between external load and TMG response. To further investigate a possible dose–response relationship of external and internal load, the external load would have to be varied systematically and TMG values would have to be collected accordingly.

Because in 3 × 3 two to three games are played per day in multiple-day tournaments [2], it is important not only to analyze a single game but also to focus on the whole tournament. Three athletes from the GC group and four athletes from the RF group were measured seven times during a two-day tournament. Neither changes between the baseline measurements on day one and day two, nor clear changes during the course of the tournament can be shown. However, there are quite high interindividual differences concerning the muscular response which might act in favor of the above-mentioned inverted-U theory.

There are some limitations regarding the practicability of the study design and the data interpretation. Since data were collected at an official tournament, the teams' main goal was to reach a successful outcome. Therefore, we aimed to conduct our measurements as quickly and accurately as possible without disturbing the athletes' usual routines. We instructed them to come to us as soon as possible after the game to collect the sensors and to carry out the TMG measurement. Also, the athletes of each team were measured in the same order on each measurement. However, in some cases, the teams used the time directly after the match to analyze the game. Then, it was not possible to capture TMG data directly after the termination of the game. For this reason, the time delay of the TMG measurement was not the same after each match. However, since the time delay was not significantly correlated with the TMG response within this analysis, we can assume that it did not influence the TMG values systematically. When the teams were not able to get to the measurement setup before the following game ended, we were not able to conduct TMG analyses for each team member. This is why the sample size of those persons for whom every measurement could be conducted is quite low. Additionally, there have been some difficulties in measuring GC. Since there have been atypical curves, challenging measurement quality, several data sets had to be excluded from analysis. Due to beforementioned time constraints, it was not possible to immediately check the data or repeat the TMG measurements during the tournament. This limits the informative value of the investigation since the sample size in GC is significantly smaller compared to RF. However, these constraints pop up in ecologically valid settings. Even though sometimes it took longer until the athletes could be measured, the time delay between the end of the game and the TMG measurement was less than 20 min. It is therefore expected that possible changes in contractility can still be measured [18, 23, 24]. Furthermore, in the context of the transferability of the research results into practice, it is questionable whether effects that show up only very shortly after the load have relevance for practice (concerning game tactics and regeneration) since the breaks between games are longer than 20 min in 3 × 3.

Although TMG measurements are sensitive to detecting changes in contractility of the muscle after specific motor tasks [19, 24, 49, 50], other authors are questioning the reliability of the method. Wiewelhove et al. [28] state that TMG markers are not sensitive enough to detect muscular performance changes after a 4-day HIT microcycle. One possible explanation might be the mechanism of fatigue, which can be peripheral (within the muscle, beyond the neuromuscular junction) or central, involving CNS and neural pathways [51]. When mainly central fatigue occurs as a result of physical exercise, peripheral mechanisms might not be impaired and, therefore, contractility of the musculature is unchanged. Especially in young athletes, higher central than peripheral fatigue was reported after repeated maximal contractions [52]. This effect might also be explained by a greater reliance on slow-twitch muscle fibers in youth athletes [53, 54]. Within this study, only adults were included, however, when analyzing the rating of perceived exhaustion, the athletes rated the games to be “hard” to “very hard”.

Within this investigation, there were no systematic changes of contractility of the musculature after a single 3 × 3 game and during the tournament. One may assume that a specific focus on muscular regeneration is not necessary to succeed in a 3 × 3 tournament in elite 3 × 3 athletes, however, there is a need to conduct more evidence based analysis on this topic to further prove it. Also, analysis of less trained athletes as well as a systematic variation of the load could be advantageous to investigate the relationships between changes in contractility following a 3 × 3 Basketball game.

## Conclusion

Three-on-three basketball is a sport with a high amount of jumps, COD, ACC, and DEC. Therefore, we hypothesized a decrease in muscular contractility due to the game load of a single 3 × 3-game. However, it seems that elite athletes are well prepared for this kind of external load in this investigation. Even though analyzes showed a significant decrease in TMG values of the GC after a single game, no systematic changes in muscular contractility with regard to the external load can be reported for RF. Concerning the course of the tournament, systematic changes can neither be reported for GC nor for RF. Because of the sport being played in tournament mode, information about the regeneration status of athletes seems to be crucial. Within this analysis, athletes were not greatly affected by game load. Therefore, one might assume that the break between games is sufficient to return to the initial level, however, this needs to be investigated in further analysis. Nevertheless, athletes report that the games are perceived as more strenuous as the tournament progresses, which could be explained by global fatigue.

## Supplementary Information

**Additional file 1.** TMG and load parameters during a 3 × 3 tournament. Dm, radial displacement of the muscle belly; Vc, contraction velocity; PL, player load; GC, M. Gastrocnemius medialis; RF, M. Rectus femoris.

## Data Availability

The datasets for this study can be found in Open Science Framework [OSF, 10.17605/OSF.IO/HXF8G; https://www.osf.io/hxf8g/].

## Abbreviations

COD: Change of direction
Dm: Maximal radial displacement
GC: M. Gastrocnemius medialis
HR: Heart rate
RF: M. Rectus femoris
SD: Standard deviation
Session RPE: Rate of perceived exhaustion per Session
TMG: Tensiomyography

## References

[1] Montgomery PG, Maloney BD (2018). Three-by-three basketball: Inertial movement and physiological demands during elite games. Int J Sports Physiol Perform.

[2] McGown RB, Ball NB, Legg JS, Mara JK (2020). The perceptual, heart rate and technical-tactical characteristics of 3 × 3 basketball. Int J Sports Sci Coach.

[3] Fox JL, Scanlan AT, Stanton R (2017). A review of player monitoring approaches in basketball: current trends and future directions. J Strength Cond Res.

[4] Halson SL (2014). Monitoring training load to understand fatigue in athletes. Sports Med.

[5] Johnston RD, Gabbett TJ, Jenkins DG, Hulin BT (2015). Influence of physical qualities on post-match fatigue in rugby league players. J Sci Med Sport.

[6] Montgomery PG, Maloney BD (2018). 3x3 basketball: performance characteristics and changes during elite tournament competition. Int J Sports Physiol Perform.

[7] Akenhead R, Nassis GP (2016). Training load and player monitoring in high-level football: current practice and perceptions. Int J Sports Phys Perform.

[8] Fields JB, Lameira DM, Short JL, Merrigan JM, Gallo S, White JB, Jones MT (2021). Relationship between external load and self-reported wellness measures across a men’s collegiate soccer preseason. J Strength Cond Res.

[9] Aoki MS, Ronda LT, Marcelino PR, Drago G, Carling C, Bradley PS, Moreira A (2017). Monitoring training loads in professional basketball players engaged in a periodized training program. J Strength Cond Res.

[10] Reina M, García-Rubio J, Ibáñez SJ (2020). Training and competition load in female basketball: a systematic review. Int J Environ Res Public Health.

[11] Stojanović E, Stojiljković N, Scanlan AT, Dalbo VJ, Berkelmans DM, Milanović Z (2018). The activity demands and physiological responses encountered during basketball match-play: a systematic review’. Sports Med.

[12] Petway AJ, Freitas TT, Calleja-González J, Medina Leal D, Alcaraz PE (2020). Training load and match-play demands in basketball based on competition level: a systematic review. Plos One..

[13] Willberg C, Wellm D, Behringer M, Zentgraf K (2022). Analyzing acute and daily load parameters in match situations—a comparison of classic and 3 x 3 basketball. Int J Sports Sci Coach.

[14] Reina Román M, García-Rubio J, Feu S, Ibáñez SJ (2019). Training and competition load monitoring and analysis of women’s amateur basketball by playing position: approach study. Front Psychol.

[15] Vanrenterghem J, Nedergaard NJ, Robinson MA, Drust B (2017). training load monitoring in team sports: a novel framework separating physiological and biomechanical load-adaptation pathways. Sports Med.

[16] Wang T, Lin Z, Day RE, Gardiner B, Landao-Bassonga E, Rubenson J, Kirk TB (2013). Programmable mechanical stimulation influences tendon homeostasis in a bioreactor system’. Biotechnol Bioeng.

[17] Šimunič B, Koren K, Rittweger J, Lazzer S, Reggiani C, Rejc E, Pišot R, Narici M, Degens H (2019). tensiomyography detects early hallmarks of bed-rest-induced atrophy before changes in muscle architecture. J Appl Physiol.

[18] Blazevich AJ, Babault N (2019). Post-activation potentiation versus post-activation performance enhancement in humans: historical perspective, underlying mechanisms, and current issues. Front Physiol.

[19] García-Manso JM, Rodríguez-Matoso D, Sarmiento S, de Saa Y, Vaamonde D, Rodríguez-Ruiz D, Da Silva-Grigoletto ME (2012). Effect of high-load and high-volume resistance exercise on the tensiomyographic twitch response of biceps brachii. J Electromyogr Kinesiol.

[20] Allen DG, Lamb GD, Westerblad H (2008). Skeletal muscle fatigue: cellular mechanisms. Physiol Rev.

[21] Hettinga FJ, Konings MJ, Cooper CE (2016). Differences in muscle oxygenation, perceived fatigue and recovery between long-track and short-track speed skating. Front Physiol.

[22] Szmedra L, Im J, Nioka S, Chance B, Rundell KW (2001). Hemoglobin/myoglobin oxygen desaturation during alpine skiing’. Med Sci Sports Exerc.

[23] García-Manso JM, Rodríguez-Ruiz D, Rodríguez-Matoso D, de Saa Y, Sarmiento S, Quiroga M (2011). Assessment of muscle fatigue after an ultra-endurance triathlon using tensiomyography (TMG). J Sports Sci.

[24] Hunter AM, Galloway SDR, Smith IJ, Tallent J, Ditroilo M, Fairweather MM, Howatson G (2012). Assessment of eccentric exercise-induced muscle damage of the elbow flexors by tensiomyography. J Electromyogr Kinesiol.

[25] Sánchez-Sánchez J, Bishop D, García-Unanue J (2018). Effect of a repeated sprint ability test on the muscle contractile properties in elite futsal players. Sci Rep.

[26] Loturco I, Gil S, Laurino CF, Roschel H, Kobal R, Cal Abad CC, Nakamura FY (2015). Differences in muscle mechanical properties between elite power and endurance athletes: a comparative study. J Strength Cond Res.

[27] Hamner SR, Seth A, Delp SL (2010). Muscle contributions to propulsion and support during running. J Biomech.

[28] Wiewelhove T, Raeder C, de Paula Simola RA, Schneider C, Döweling A, Ferrauti A (2017). Tensiomyographic markers are not sensitive for monitoring muscle fatigue in elite youth athletes: a pilot study. Front Physiol.

[29] Iacono AD, Eliakim A, Meckel Y (2015). Improving fitness of elite handball players: small-sided games vs. high-intensity intermittent training. J Strength Cond Res.

[30] Borg E, Kaijser L (2006). A comparison between three rating scales for perceived exertion and two different work tests. Scand J Med Sci Sports.

[31] Macgregor LJ, Hunter AM, Orizio C, Fairweather MM, Ditroilo M (2018). Assessment of skeletal muscle contractile properties by radial displacement: the case for tensiomyography’. Sports Med.

[32] Dahmane R, Valenčič V, Knez N, Eržen I (2001). Evaluation of the ability to make non-invasive estimation of muscle contractile properties on the basis of the muscle belly response. Med Biol Eng Comput.

[33] Perotto A, Delagi EF (2011). Anatomical guide for the electromyographer: the limbs and trunk.

[34] Ditroilo M, Hunter AM, Haslam S, De Vito G (2011). The effectiveness of two novel techniques in establishing the mechanical and contractile responses of biceps femoris. Physiol Meas.

[35] Šimunič B (2012). Between-day reliability of a method for non-invasive estimation of muscle composition. J Electromyogr Kinesiol.

[36] Piqueras-Sanchiz F, Martín-Rodríguez S, Pareja-Blanco F, Baraja-Vegas L, Blázquez-Fernández J, Bautista IJ, García-García Ó (2020). Mechanomyographic measures of muscle contractile properties are influenced by electrode size and stimulation pulse duration. Sci Rep.

[37] Simola P, de Rauno Á, Raeder C, Wiewelhove T, Kellmann M, Meyer T, Pfeiffer M, Ferrauti A (2016). Muscle mechanical properties of strength and endurance athletes and changes after one week of intensive training. J Electromyogr Kinesiol.

[38] Enoka RM (1996). Eccentric contractions require unique activation strategies by the nervous system. J Appl Physiol.

[39] Dahmane R, Djordjevič S, Šimunič B, Valenčič V (2005). Spatial fiber type distribution in normal human muscle. J Biomech.

[40] Staron RS, Hagerman FC, Hikida RS, Murray TF, Hostler DP, Crill MT, Ragg KE, Toma K (2000). Fiber type composition of the vastus lateralis muscle of young men and women. J Histochem Cytochem.

[41] Pierrynowski MR, Morrison JB (1985). A physiological model for the evaluation of muscular forces in human locomotion: theoretical aspects. Math Biosci.

[42] Gardetto PR, Schluter JM, Fitts RH (1989). Contractile function of single muscle fibers after hindlimb suspension. J Appl Physiol.

[43] Metzger JM, Moss RL (1990). Calcium-sensitive cross-bridge transitions in mammalian fast and slow skeletal muscle fibers. Science.

[44] Moore RL, Stull JT (1984). Myosin light chain phosphorylation in fast and slow skeletal muscles in situ. Am J Physiol Cell Physiol.

[45] Rassier DE, MacIntosh BR (2000). Coexistence of potentiation and fatigue in skeletal muscle. Braz J Med Biol Res.

[46] Gil S, Loturco I, Tricoli V, Urgrinowitsch C, Kobal R, Abad CCC, Roschel H (2015). Tensiomyographiy parameters and jumping and sprinting performance in Brazilian elite soccer players. Sports Biomech.

[47] García-García O, Cuba-Dorado A, Álvarez-Yates T, Carballo-López J, Iglesias-Caamaño M (2019). Clinical utility of tensiomyography for muscle function analysis in athletes. Open Access J Sports Med..

[48] Peterson KD, Quiggle GT (2016). Tensiomyographical response to accelerometer loads in female collegiate basketball players. J Sports Sci.

[49] Simola P, de Rauno Á, Harms N, Raeder C, Kellmann M, Meyer T, Pfeiffer M, Ferrauti A (2015). Assessment of neuromuscular function after different strength training protocols using tensiomyography. J Strength Cond Res.

[50] Wiewelhove T, Raeder C, Meyer T, Kellmann M, Pfeiffer M, Ferrauti A (2015). Markers for routine assessment of fatigue and recovery in male and female team sport athletes during high-intensity interval training. PLoS ONE.

[51] Gandevia SC (2001). Spinal and supraspinal factors in human muscle fatigue. Physiol Rev.

[52] Ratel S, Kluka V, Garcia Vicencio S, Jegu A-G, Cardenoux C, Morio C, Coudeyre E, Martin V (2015). Insights into the mechanisms of neuromuscular fatigue in boys and men. Med Sci Sports Exerc.

[53] Chen TC, Chen H-L, Liu Y-C, Nosaka K (2014). Eccentric exercise-induced muscle damage of pre-adolescent and adolescent boys in comparison to young men. Eur J Appl Physiol.

[54] Marginson V, Rowlands AV, Gleeson NP, Eston RG (2005). Comparison of the symptoms of exercise-induced muscle damage after an initial and repeated bout of plyometric exercise in men and boys. J Appl Physiol.

