# Supplementary figures and images for: The relationship between external and internal load parameters in 3 × 3 basketball tournaments
Source: BMC Sports Sci Med Rehabil. 2022 Aug 3;14:152. doi: 10.1186/s13102-022-00530-1 (PMC9351101; doi:10.1186/s13102-022-00530-1)

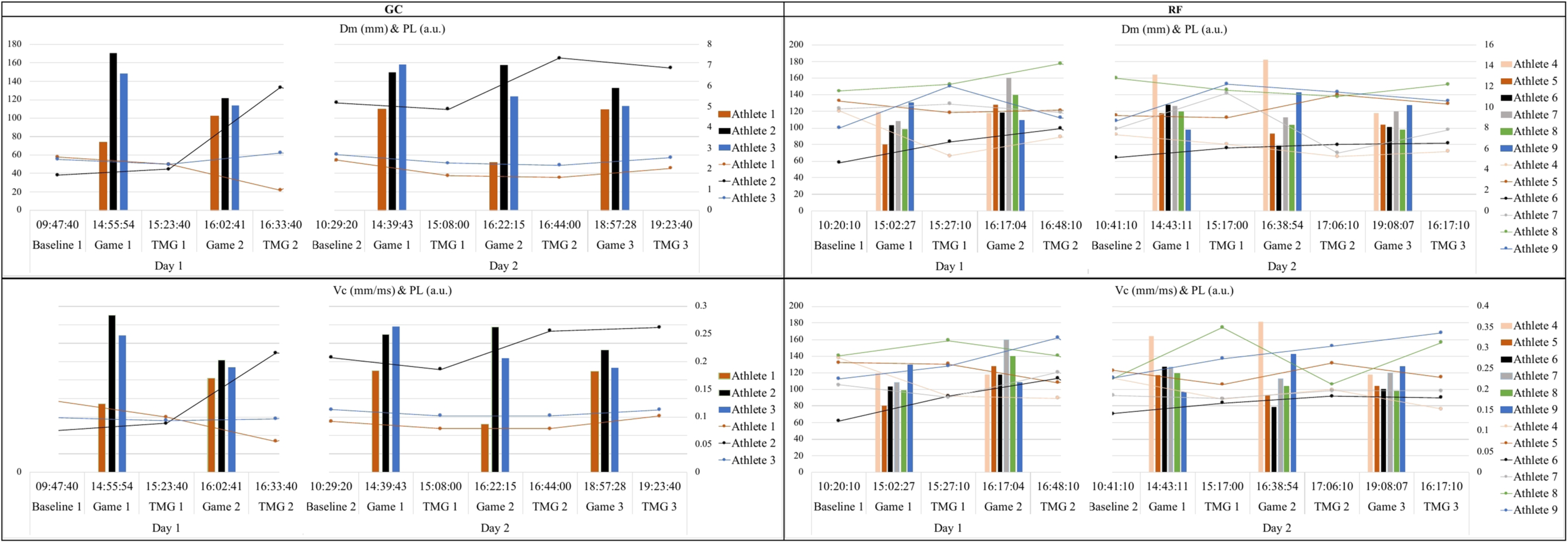

Supplement: Supplementary file 1 — Additional file 1. TMG and load parameters during a 3 × 3 tournament. Dm, radial displacement of the muscle belly; Vc, contraction velocity; PL, player load; GC, M. Gastrocnemius medialis; RF, M. Rectus femoris. [file 13102_2022_530_MOESM1_ESM.tif]
